# Added value of non-rigid image registration for intrafraction dose accumulation in magnetic resonance imaging-guided prostate radiotherapy

**DOI:** 10.1016/j.phro.2025.100711

**Published:** 2025-01-31

**Authors:** Georgios Tsekas, Cornel Zachiu, Gijsbert H. Bol, Johannes C.J. de Boer, Bas W. Raaymakers

**Affiliations:** Department of Radiotherapy, University Medical Center Utrecht, Heidelberglaan 100, 3584 CX, Utrecht, The Netherlands

**Keywords:** Prostate cancer, Adaptive radiotherapy, Deformable image registration, Rigid image registration, Intrafraction motion, Hypofractionated radiotherapy

## Abstract

This work investigates potential advantages of non-rigid versus rigid image registration for intrafraction dose reconstruction in hypofractionated prostate radiotherapy. The data of 15 patients were analyzed using 3D cine magnetic resonance imaging (MRI) in combination with machine log files and the accumulated dose distributions were compared to the planned ones. Both image registration methods resulted in comparable results for the majority ( ∼ 95%) of patient fractions. However, better image alignment was reported for the non-rigid method compared to rigid in cases of transient gas pockets, indicating better image registration quality in the presence of large intrafraction deformations.

## Highlights

• The added value of non-rigid dose accumulation in prostate radiotherapy was explored.
• Rigid and non-rigid image registration methods were compared.
• Dose accumulation was performed using 3D cine MRI scans combined with machine log files.
• Both rigid and deformable dose accumulation had similar reconstructed dose results.
• Non-rigid dose accumulation was better in cases of large intrafraction motion.

## 1 Introduction

Intrafraction motion originating from anatomical and physiological processes can pose a challenge in magnetic resonance imaging (MRI)-guided radiotherapy of prostate cancer patients and as a result, the delivered dose might differ from the intended one. Therefore, accurate dose accumulation methods are necessary in order to better correlate clinical outcomes to delivered treatment plans and to enable adaptive plan optimization based on the reconstructed dose [1]. A variety of dose reconstruction methods have been explored in literature [2], [3], [4], [5]. In the context of dose accumulation, image registration plays an essential role in estimating anatomical motion. While rigid image registration (RIR) methods merely rely on translations and rotations to reconstruct the delivered dose, deformable image registration (DIR) methods can offer more in-depth, voxel-wise information about the underlying anatomical deformations and are widely available.

DIR is particularly interesting for prostate radiotherapy, where large deformations can happen at an intrafraction time-scale [6], [7], [8]. While RIR could suffice in explaining the prostate intrafraction motion, since the prostate body remains relatively rigid and does not undergo significant deformations, the surrounding organs-at-risk (OAR), such as the bladder and the rectum, can considerably deform.

The feasibility of intrafraction dose accumulation using online MRI in combination with machine log files has already been explored for prostate cancer patients [9], [10]. Significant efforts are being made towards deformable dose accumulation during prostate cancer treatments, however no systematic study has been conducted to evaluate the actual benefits provided of DIR, compared to RIR. Additionally, the majority of currently employed clinical workflows merely rely on rigid adaptations, thus not accounting for non-rigid intrafraction deformations of the bladder and the rectum.

This work explores the added value of DIR for intrafraction dose accumulation in hypofractionated MRI-guided prostate radiotherapy. The outcomes of this study can provide insights into the benefits provided by DIR relative to RIR and to what extent deformable dose accumulation is necessary for this task.

## 2 Materials and methods

### 2.1 Clinical workflow

A total of 71 fractions obtained from 15 patients treated with a 5 × 7.25 Gy scheme for low and intermediate-risk prostate cancer were included in this study. The study was conducted in accordance with the declaration of Helsinki: all patients signed an informed consent to participate in the MOMENTUM (NCT04075305) and/or UPC (NCT04228211) studies. Four patient fractions were dropped due to potentially corrupted machine log files. The analyzed patients were treated with an online adaptive workflow on the 1.5 T MR-linac [11], where a daily T2-weighted (T2w) MRI scan was acquired and was followed by a position verification (PV) T2w scan to account for intrafraction motion by means of a rigid plan shift. During beam-on time, 3D balanced turbo-field echo (bTFE) cine MRI data were acquired every 9.36 s, with the mean duration of 3D cine MRI scans being 9 min corresponding to 57 dynamics on average (Supplementary Figure S1). An overview of the scan parameters of the different MRI sequences can be found in Supplementary Table S1.

**Fig. 1:**
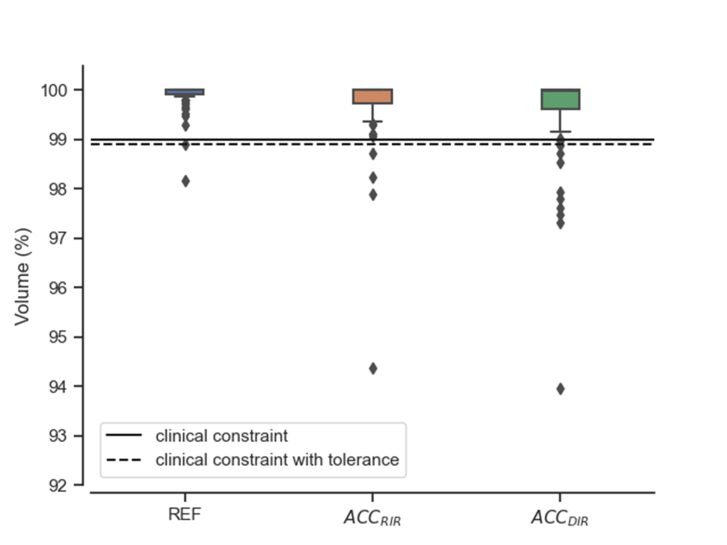
Planned (REF) versus accumulated (ACC) dose for the V_95%_ of the CTV for all patient fractions. The REF column corresponds to the reference/planned dose while ACC_RIR_ and ACC_DIR_ show the accumulated dose distributions using RIR and DIR respectively.

### 2.2 Dose accumulation

In order to perform dose accumulation, the 3D cine MRI scans were used in combination with linac-exported machine log files to calculate the delivered dose at each MRI timestamp (every 9.36 s). The exported machine log files describe the machine output and can be retrospectively used to reconstruct the delivered dose distribution [9]: The reference dose distribution was divided into partial dose files, based on the timestamps of the cine MRI instances and the machine states, followed by image registration (between the cine MRI instances and the reference T2w PV MRI) and finally the partial dose distributions were warped and summed using the calculated transformations.

The clinically approved dose distribution prior to delivery, with the daily contours adjusted to match the online PV anatomy, was considered the reference dose (REF). Two image registration methods were used to accumulate the dose: a RIR method using translations and rotations and a DIR algorithm. For RIR the elastix package was used [12], [13], based on the same settings of previous relevant work for intrafraction prostate tracking [6]. An isotropic expansion of 2 cm was applied to the clinical tumour volume (CTV) and the CTV＋2cm mask was used for estimating the motion [6]. DIR was performed using Evolution [14], [15], for the cross-contrast image registration (from T2w to bTFE), and an optical flow-based algorithm [16] for the image registration between different instances of the 3D bTFE cine MRI (Supplementary Figure S2).

The total transformation for each instance of the 3D bTFE cine MRI using RIR was calculated with respect to the PV reference scan. For DIR the total deformation vector fields (DVF) were accumulated as following [17]:

(1) $$DVF_{{\text{total}}_{\text{i}}}=DVF_{\text{PV}}⊕DVF_{{\text{cine MRI}}_{\text{i}}},$$

where DVF_PV_ was the DVF describing the deformation between the PV MRI and the first reference 3D cine MRI instance and DVF_cine MRI_i__ the deformations between the ith 3D cine MRI instance and the first reference 3D cine MRI instance of the beam-on imaging (Supplementary Figure S2).

### 2.3 Analysis

The accumulated (ACC) dose distributions were evaluated using the reference contours (PV anatomy) and the clinical plan parameters. Therefore, the V_95%_ of the CTV, D_5cc_ of the bladder and D_1cc_ of the rectum were calculated. In order to assess the over-/underdosage in the OARs, the dosimetric differences for the D_5cc_ of the bladder and D_1cc_ of the rectum between the REF and ACC dose distributions were reported for the two different methods. For assessing the quality of the registration, the mean structural similarity index (SSIM) was calculated for all voxels inside the CTV＋2cm mask. Furthermore, the jacobian of the DVFs was calculated to evaluate the anatomical plausibility of the results of DIR.

## 3 Results

Fig. 1 presents the results for the CTV coverage using the two different dose accumulation methods. In the majority of the cases both methods resulted in comparable accumulated dose. RIR-based dose accumulation reported CTV coverage lower than the clinical constraints in 6% of all fractions (4/71 fractions). On the contrary, DIR-based dose accumulation flagged an additional four patient fractions (8/71 fractions) for sub-optimal CTV V_95%_ coverage (11% out of the total fractions). Concerning the OAR, higher reconstructed dose was reported in some fractions, however the values were still within the clinical constraints. (Supplementary Figure S3).

**Fig. 2:**
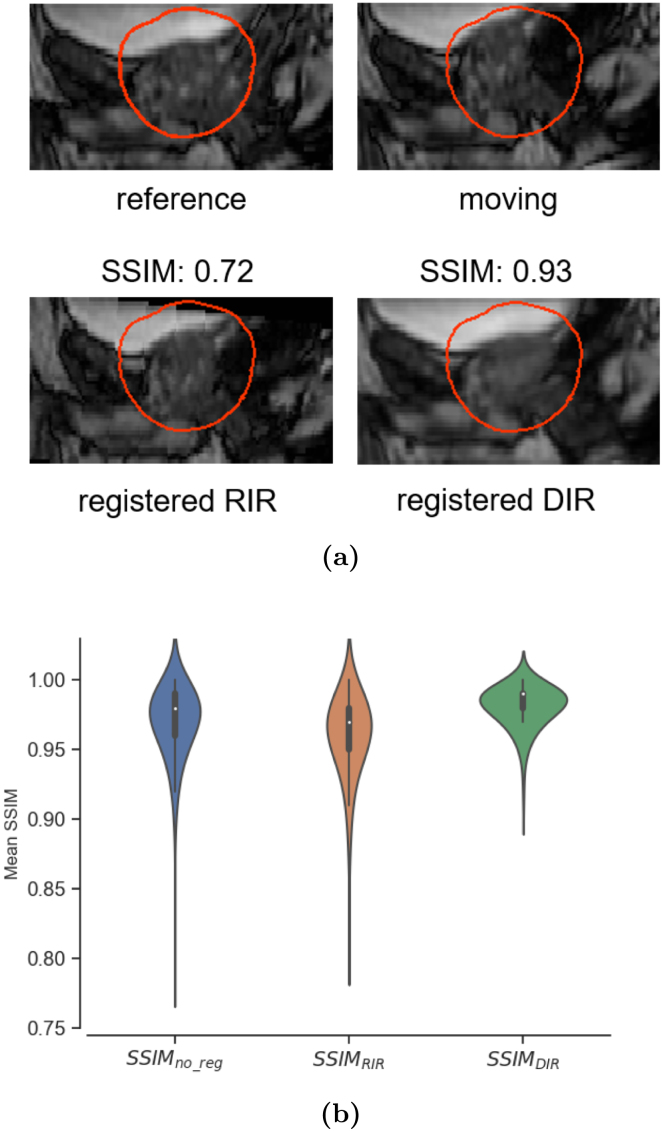
Evaluation of post registration image quality. (a) An example of large intrafraction deformation between the reference and moving 3D cine MRI instances due to the presence of a gas pocket. The mean SSIM, calculated for all voxels inside the CTV＋2cm mask (shown in red), is presented alongside the corresponding registered image for the RIR and DIR methods. (b) Mean SSIM, calculated for the CTV＋2cm mask, for all 3D cine MRI instances before registration (SSIM_no_reg_), after RIR and DIR. Each sample corresponds to the mean SSIM of every registered cine MRI instance to the reference (first) 3D cine MRI instance for each patient fraction. (For interpretation of the references to color in this figure legend, the reader is referred to the web version of this article.)

Additional inspection in the patient fractions with low CTV dose revealed large intrafraction motion events. An example of a gas pocket present in the rectum is presented on Fig. 2a. The differences in mean SSIM for the registered 3D cine MRI images for all patient fractions using the two methods are presented on Fig. 2b. Overall, improvements of the mean SSIM inside the CTV＋2cm mask for the DIR-based warped images were observed. The 5th- and 95th percentile values of the mean SSIM were 0.8 and 0.9 before registration, 0.85 and 0.88 after RIR, and 0.91 and 0.93 after DIR respectively. The voxel-wise dosimetric differences between the REF and ACC dose distributions for the same fraction presented in Fig. 2a are shown on Supplementary Figure S4.

Furthermore, the mean values of the jacobian determinant inside the prostate contour for all registered 3D cine MRI images resulted in an average of 1.00 ± 0.02, while negative values were not present.

## 4 Discussion

This work presents a comparison of rigid and deformable image registration methods for intrafraction dose accumulation of prostate cancer patients on the 1.5 T MR-linac. Both RIR and DIR techniques were compared and the added value of DIR was investigated. Overall, small differences were observed in the majority of accumulated dose distributions between the different methods for the analyzed data. Nonetheless, the rigid approach was not able to capture the deformations of the OARs, particularly for the rectum, where transient gas pockets led to notable deformations.

Compared to existing literature which was limited to investigating the use of machine log files for rigid intrafraction dose reconstruction [9], [10], we present the added value of DIR for intrafraction dose accumulation in hypofractionated prostate radiotherapy. Both RIR- and DIR-based methods resulted in comparable results for the targets and OAR coverage using the clinical plan parameters. Our findings indicate that despite large deformations and the limited ability of the rigid method to capture them for the bladder and rectum, this had no significant impact on fulfilling the clinical constraints. Additional analysis of the dose differences, in the cases for which low reconstructed target dose was reported, did not result in a violation of the overdose constraints in the OARs.

While similar dosimetric coverage was reported for the majority of the cases, DIR resulted in higher SSIM and was able to capture transient events, such as gas pockets, effectively (Fig. 2). SSIM was chosen as a quality assurance (QA) metric due to previous studies demonstrating that it had a high correlation with registration errors out of a wide selection of intensity-based criteria for image registration [18]. The high mean SSIM values before registration show that the majority of samples had very little motion. Nonetheless, it is evident that with image registration, the outliers improve with both RIR and DIR. Additionally, the values of the jacobian determinant of the DVFs inside the prostate contour were non-negative and correspond to near-incompressible prostate throughout the treatment fraction, indicating that the DIR estimated anatomically feasible deformations.

In terms of algorithm selection, for rigid dose accumulation, elastix was used due to its robustness and to its previously demonstrated performance in rigid dose accumulation [5], [9]. For deformable dose accumulation, a combination of an optical flow-based algorithm [16] and the Evolution algorithm [14], [15] were used. Both algorithms have demonstrated clinically-acceptable performance in deformable image registration for a variety of applications in the scope of image-guided ratiotherapy [19], [20], [21], [22]. While the employment of alternative dose accumulation algorithms is generally not expected to result in identical results, the differences between different methods with a clinically-acceptable precision and accuracy are not expected to be clinically relevant [20], [22]. In addition, as discussed by previous studies, the used algorithms are robust to minor changes in scan parameters, although significant changes in the MRI acquisition sequences might lead to a need for re-configuration of the algorithms prior to use.

In this work, intrafraction dose reconstruction was performed for a hypofractionated treatment workflow. Recent clinical approaches move towards ultra hypofractionated treatments of only 2 fractions (HERMES, TURBO trials) by using a sub-fractionated clinical workflow where online rigid plan shifts aim to account for intrafraction motion, without the need for dose accumulation [21]. The results of this study suggest that rigid corrections can explain the majority of intrafraction motion events and could thus be potentially used for ultra hypofractionated treatments. Additionally, the duration of beam-on data used for dose accumulation of this study (9 min on average) is comparable to typical beam-on times in a clinical setup.

The decision of using non-rigid over rigid image registration for dose accumulation in prostate cancer patients is a trade-off between accuracy and complexity. While the results of RIR (translations and rotations) are easier to interpret, rigid methods are inherently limited in cases with large intrafraction deformations, such as bladder filling and gas pockets in the rectum. DIR on the other hand, can successfully capture non-rigid anatomical deformations, but it typically results in longer calculation times, the DVF outputs are difficult to interpret and require extensive QA [22], [23]. Therefore, the choice of a dose accumulation method should be made based on the ultimate goal of the analysis. If the intention is to extract dose–effect relationships that describe toxicity, DIR would be the most accurate way to establish the delivered dose. Additionally, DIR can enable the use of dose accumulation for plan re-optimization in online adaptive stop-go treatments. Nonetheless, RIR could still provide a good solution for online plan re-optimizations for intrafraction motion management. The validation of this data is still ongoing and future work will focus on correlating RIR- and DIR-based accumulated dose with treatment outcomes.

In conclusion, both rigid- and non-rigid image registration-based methods reported comparable results for the intrafraction dose reconstruction of hypofractionated prostate cancer patients on the 1.5 T MR-linac. RIR failed to describe sudden non-rigid motion effects, but without resulting in large dosimetric differences. On the other hand, DIR can be of added value if used for extracting dose–effects relationships and can enhance the precision of online adaptive re-planning in MRI-guided prostate radiotherapy.

## CRediT authorship contribution statement

**Georgios Tsekas:** Conceptualization, Methodology, Software, Formal analysis, Writing – original draft. **Cornel Zachiu:** Conceptualization, Methodology, Software, Writing – review & editing. **Gijsbert H. Bol:** Conceptualization, Methodology, Writing – review & editing. **Johannes C.J. de Boer:** Conceptualization, Methodology, Writing – review & editing. **Bas W. Raaymakers:** Conceptualization, Methodology, Writing – review & editing.

## Declaration of competing interest

The authors declare that they have no known competing financial interests or personal relationships that could have appeared to influence the work reported in this paper.

## Appendix A Supplementary data

MMC S1 Supplementary Material.
