# Supplementary material for: Added value of non-rigid image registration for intrafraction dose accumulation in magnetic resonance imaging-guided prostate radiotherapy
Source: Phys Imaging Radiat Oncol. 2025 Jan 31;33:100711. doi: 10.1016/j.phro.2025.100711 (PMC11868999; doi:10.1016/j.phro.2025.100711)
Supplement: MMC S1 — Supplementary Material. [file mmc1.pdf]

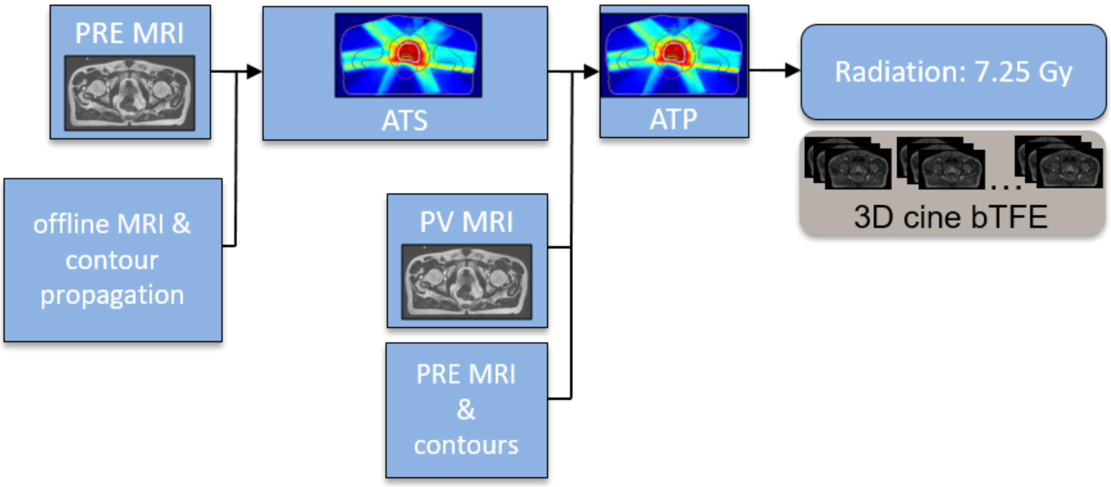

Figure S1: The MR-guided online workflow for 5x7.25 Gy prostate cancer patients used in this study. Parallel to beam-on, 3D bTFE cine MRI data were acquired every 9.36 seconds. Abbreviations: PRE: pre-treatment, PV: position verification, ATS: Adapt-to-shape, ATP: Adapt-to-position.

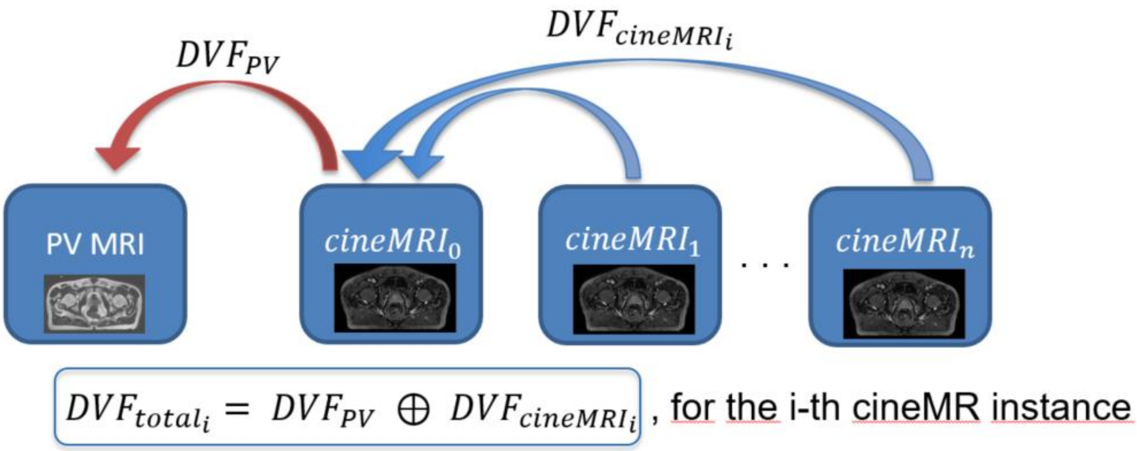

Figure S2: Overview of the DIR dose accumulation workflow. Initially DVF_{PV} was estimated using Evolution (red arrow). Then, for each cine MRI instance, the individual $DVF_{cineMRI_i}$ were calculated using an optical flow-based algorithm (blue arrows). Finally, the total DVF for each cine MRI was calculated by combining DVF_{PV} and $DVF_{cineMRI_i}$.

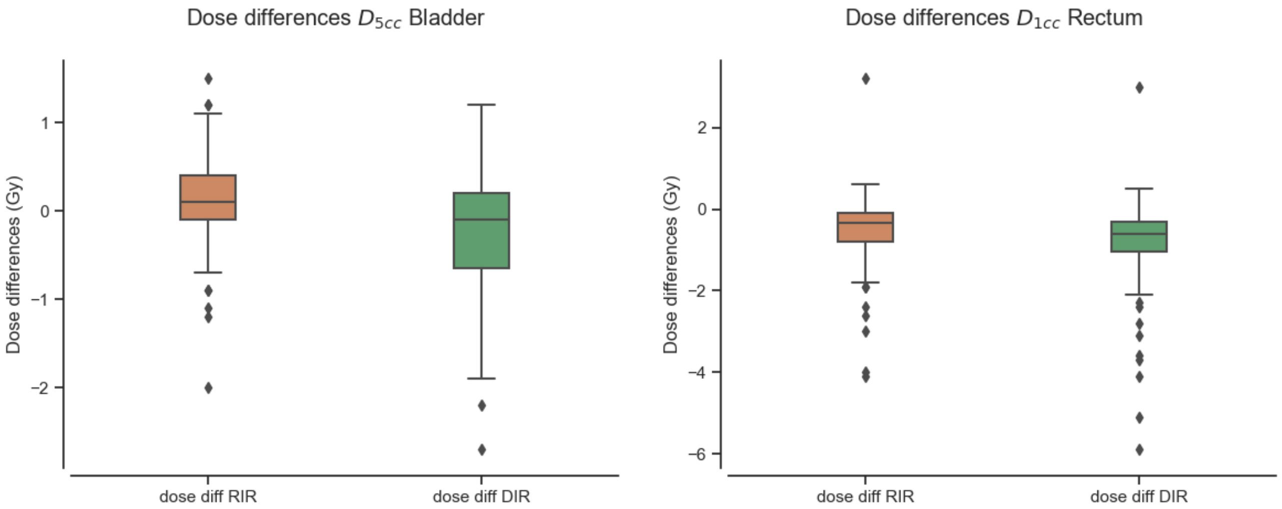

Figure S3: Signed dose differences between accumulated and reference dose (ACC-REF) for the D_{5cc} of the bladder and D_{1cc} of the rectum using rigid and deformable image registration methods. Positive values indicate an increase in the accumulated dose compared to the planned one.

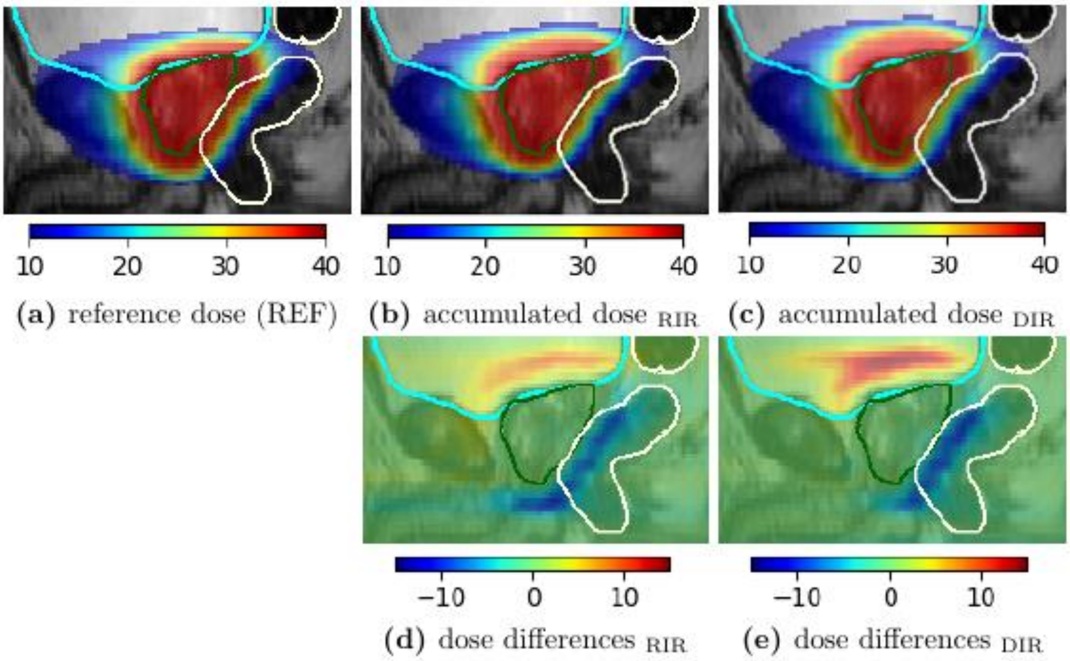

Figure S4: An example of the differences of the accumulated dose distributions for a case with large intrafraction deformations. The planned reference (REF) dose is presented next to the RIR- and DIR-based dose accumulation (a-c). All dose distributions are overlaid on the reference MRI anatomy and the bladder (cyan), CTV (green) and rectum (white) contours are visible on the sagittal slices. Additionally, the signed dose differences (ACC-REF) between the accumulated and the planned dose files are presented (d-e).

Description	bTFE 3D cine MRI	T2w 3D
Repetition time (TR)	4.7 ms	1635 ms
Echo time (TE)	2.3 ms	120 ms
Flip angle	50 degrees	90 degrees
Acquisition voxel spacing	2 x 2 x 2.2 mm	1.5 x 1.5 x 2 mm
Reconstructed voxel spacing	1 x 1 x 2.2 mm	0.76 x 0.76 x 2 mm

Table S1: Overview of the scan parameters for the different MRI sequences
